# Isoprenoid Pyrophosphate-Dependent Transcriptional Regulation of Carotenogenesis in *Corynebacterium glutamicum*

**DOI:** 10.3389/fmicb.2017.00633

**Published:** 2017-04-24

**Authors:** Nadja A. Henke, Sabine A. E. Heider, Silvin Hannibal, Volker F. Wendisch, Petra Peters-Wendisch

**Affiliations:** Genetics of Prokaryotes, Faculty of Biology, Center for Biotechnology, Bielefeld UniversityBielefeld, Germany

**Keywords:** regulation of carotenogenesis, terpenoid biosynthesis, actinobacteria, isoprenoid pyrophosphates as inducers, CrtR, MarR-type regulators

## Abstract

*Corynebacterium glutamicum* is a natural producer of the C50 carotenoid decaprenoxanthin. The *crtEcg0722crtBIYEb* operon comprises most of its genes for terpenoid biosynthesis. The MarR-type regulator encoded upstream and in divergent orientation of the carotenoid biosynthesis operon has not yet been characterized. This regulator, named CrtR in this study, is encoded in many actinobacterial genomes co-occurring with terpenoid biosynthesis genes. CrtR was shown to repress the *crt* operon of *C. glutamicum* since DNA microarray experiments revealed that transcript levels of *crt* operon genes were increased 10 to 70-fold in its absence. Transcriptional fusions of a promoter-less *gfp* gene with the *crt* operon and *crtR* promoters confirmed that CrtR represses its own gene and the *crt* operon. Gel mobility shift assays with purified His-tagged CrtR showed that CrtR binds to a region overlapping with the −10 and −35 promoter sequences of the *crt* operon. Isoprenoid pyrophosphates interfered with binding of CrtR to its target DNA, a so far unknown mechanism for regulation of carotenogenesis. The molecular details of protein-ligand interactions remain to be studied. Decaprenoxanthin synthesis by *C. glutamicum* wild type was enhanced 10 to 30-fold upon deletion of *crtR* and was decreased 5 to 6-fold as result of *crtR* overexpression. Moreover, deletion of *crtR* was shown as metabolic engineering strategy to improve production of native and non-native carotenoids including lycopene, β-carotene, C.p. 450 and sarcinaxanthin.

## Introduction

Carotenoids, natural yellow to red colored pigments, are the colorful representatives of the versatile and extensive group of terpenoids. Carotenoids have diverse biological functions serving for instance as photo protectors, light harvesting molecules or as membrane stabilizers (Sandoval et al., 1984; Lee and Schmidt-Dannert, 2002). They are synthesized by plants, fungi, algae and bacteria (Britton et al., 2004). Commercially, carotenoids and their derivatives are mainly applied in the food, beverage and cosmetics industries (Downham and Collins, 2000; Winterhalter and Rouseff, 2001; Dembitsky, 2005), but they are also receiving increasing attention due to their potential beneficial effects on human health (Armstrong, 1994; Sandmann, 2001; Umeno et al., 2005; Das et al., 2007). The total commercial market value of carotenoids was $1.5 billion in 2014 and it is expected to increase to $1.8 billion in 2019 (BBCResearch, 2015).

*Corynebacterium glutamicum*, a GRAS organism used in industrial biotechnology, is a natural producer of the C50 carotenoid decaprenoxanthin, responsible for its characteristic yellow pigmentation. *C. glutamicum* belongs to a rare group of bacteria producing long chain carotenoids. Carotenogenesis is not essential in *C. glutamicum* since transposon mutants (Krubasik et al., 2001a,b; Sandmann and Yukawa, 2005) as well as directed gene deletion mutants were shown to be deficient of carotenoid biosynthesis, but viable (Heider et al., 2012). C50 carotenoids have been mainly isolated from extremely halophilic archaea (Kelly and Jensen, 1967; Pfander, 1994) and from Gram-positive bacteria of the order *Actinomycetales* (Netzer et al., 2010). The pathway for decaprenoxanthin biosynthesis starting from the precursors isopentenyl pyrophosphate (IPP) and dimethylallyl pyrophosphate (DMAPP) and the respective carotenogenic genes of *C. glutamicum* are characterized (Krubasik et al., 2001a; Heider et al., 2012). Besides the biosynthesis of the ε-cyclic decaprenoxanthin in *C. glutamicum* only four other pathways toward C50 carotenoids have been elucidated: the pathway of the β-cyclic C.p.450, first isolated from *C. poinsettiae* (Norgård et al., 1970), in *Dietzia* sp. CQ4 (Tao et al., 2007), the γ-cyclic C50 carotenoid sarcinaxanthin in *Micrococcus luteus* NCTC2665 (Netzer et al., 2010) and recently the route to the acyclic bacterioruberin in the extremely halophilic archaeon *Haloarcula japonica* was described (Yang et al., 2015).

In many bacteria the carotenogenic genes are clustered. *C. glutamicum* possesses the *crt* operon with seven co-transcribed genes (*crtE*, cg0722, *crtB, crtI, crtY*_*e*_, *crtY*_*f*_, *crtEb*) necessary for the conversion of IPP and DMAPP to decaprenoxanthin, and one gene of unknown function (cg0722). A second functional phytoene synthase is encoded elsewhere in the genome (Heider et al., 2012). The major geranylgeranyl pyrophosphate (GGPP) synthase of *C. glutamicum* is not encoded by *crtE*, the first gene of the *crt* operon, but by *idsA* that is located elsewhere on the *C. glutamicum* chromosome (Heider et al., 2014a). It is not known whether synthesis of the redundant GGPP synthase and phytoene synthase enzymes is regulated.

Biosynthesis of terpenoids is commonly triggered by photo-oxidative stress factors in plant, fungi and bacteria (Bramley and Mackenzie, 1988), as they serve e.g., as light harvesting molecules or as protection against excess light energy or against the resulting reactive oxygen species (Johnson and Schroeder, 1996; Britton, 2008). Little is known about the regulatory mechanisms governing carotenoid biosynthesis in bacteria which may change in response to blue light, oxygen levels or growth phase (Johnson and Schroeder, 1996). The mechanism of light-induced carotenogenesis in the non-photosynthetic Gram-negative bacterium *Myxococcus xanthus* (Fontes et al., 2003), the Gram-positive bacteria *Streptomyces coelicolor* A3(2) (Takano et al., 2005), *Thermus thermophilus* (Takano, 2016) and *Bacillus megaterium* (Takano et al., 2015b) has been studied in more detail. MerR-type transcriptional regulators with a vitamin B12 binding domain are involved in regulation of carotenogenesis in those organisms. In addition, the RNA polymerase sigma factor SigF has been shown to be involved in regulation of carotenoid biosynthesis in *Mycobacterium smegmatis* (Provvedi et al., 2008).

Regulation of carotenoid biosynthesis in *C. glutamicum* has not been investigated so far. Gene expression analyses performed under different stress conditions revealed that mRNA levels of *crtE*, the first gene of the *crt* operon, altered in response to various stresses (Silberbach et al., 2005; Follmann et al., 2009; Jochmann et al., 2009). The putative multiple antibiotic resistance (MarR)-family transcriptional regulator gene cg0725, named *crtR* due to the results described in this study, is located upstream of the *crt* operon and is transcribed in divergent orientation to the *crt* operon. Previously, it has been shown that *crtR* was disrupted by transposon insertion in a mutant accumulating 3-fold more decaprenoxanthin (Krubasik et al., 2001a), but the regulatory mechanism has not been characterized. The majority of characterized MarR proteins are transcriptional repressors and their genes are generally found in the vicinity or are part of the regulated gene cluster (Wilkinson and Grove, 2006). This genetic organization and the finding that transposon insertions into this gene and the orthologuous gene in the related *Mycobacterium marinum* increased biosynthesis of decaprenoxanthin and β-carotene (Krubasik et al., 2001a; Gao et al., 2003), prompted us to characterize CrtR with respect to regulation of carotenogenesis in *C. glutamicum*.

## Materials and methods

### Bacterial strains, media, and growth conditions

The strains and plasmids used in this work are listed in Table 1. *C. glutamicum* ATCC 13032 was used as wild type (WT) and the prophage cured *C. glutamicum* MB001 (Baumgart et al., 2013) as basal strain for genetic engineering. *C. glutamicum* cultivations were performed in CGXII medium with 100 mM glucose as carbon and energy source (Eggeling and Reyes, 2005) after inoculation to an initial OD_600_ of 1 at 30°C in either a volume of 50 ml in 500 ml flasks with two baffles shaking at 120 rpm or in 1 ml volume in microtiterplates at 1100 rpm at 30°C using Biolector® micro fermentation system (m2p-labs GmbH, Baesweiler, Germany). The growth was followed by measuring the OD_600_ using a Shimadzu UV-1202 spectrophotometer (Duisburg, Germany). Cloning was conducted with *E. coli* DH5α as host, cultivated in LB medium at 37°C. When appropriate, kanamycin (25 μg/ml), spectinomycin (100 μg/ml), or ampicillin (100 μg/ml) was added to the culture medium. If not stated otherwise 1 mM of IPTG was added for induction of gene expression at inoculation of the main culture.

**Table 1:** **Strains, plasmids and oligonucleotides used in this study**.

**Strain, plasmid or oligonucleotide**	**Relevant characteristics or sequence**	**References**
***E. coli* STRAINS**		
DH5α	F^−^*thi*-1 *endA1 hsdR17*(r^−^ m^−^) *supE44* Δ*lacU169* (ϕ80*lacZ*ΔM15) *recA1 gyrA96 relA1*	Hanahan, 1983
BL21 (DE3)	F^−^*ompT hsdSB*(${\text{r}}_{B}^{-}$ ${\text{m}}_{B}^{-}$) *gal dcm* (DE3)	Novagen
***C. glutamicum*** **STRAINS**		
WT	ATCC 13032	Abe et al., 1967
WTΔ*crtR*	cg0725 (*crtR*) deletion mutant of *C. glutamicum* ATCC 13032	this work
MB001	ATCC 13032 with in-frame deletion of prophages cgp1 (cg1507-cg1524), cgp2 (cg1746-cg1752), and cgp3 (cg1890-cg2071)	Baumgart et al., 2013
MB001Δ*crtR*	cg0725 (*crtR*) deletion mutant of *C. glutamicum* MB001	this work
LYC5	*crtY_*e*_Y_*f*_Eb* deletion mutant of MB001 with artificial operon containing *crtE, crtB* and *crtI* under control of the P*_*tuf*_* promoter integrated into the chromosome and chromosomal promoter exchange of *dxs* (P*_*tuf*_*)	Henke et al., 2016
LYC5Δ*crtR*	cg0725 (*crtR*) deletion mutant of *C. glutamicum* LYC5	this work
BETA3	LYC5 derivative with *crtY_*Pa*_* under control of the P*_*tuf*_* promoter integrated into the chromosome	Henke et al., 2016
BETA3 Δ*crtR*	cg0725 (*crtR*) deletion mutant of *C. glutamicum* BETA3	this work
**PLASMIDS**		
pK19*mobsacB*	Km^R^; *E. coli*/*C. glutamicum* shuttle vector for construction of insertion and deletion mutants in *C. glutamicum* (pK18 *oriV_*Ec*_ sacB lacZ*α)	Schäfer et al., 1993
pK19*mobsacB*-*crtR*	pK19*mobsacB* with a cg0725 deletion construct	Henke et al., 2016
pVWEx1	Km^R^; *E. coli*/*C. glutamicum* shuttle vector for regulated gene expression (P_tac_, *lacI*^q^, pCG1 *oriV_*Cg*_*)	Peters-Wendisch et al., 2001
pVWEx1-*crtR*	pVWEx1 derivative for IPTG-inducible expression of cg0725 from *C. glutamicum* containing an artificial ribosome binding site in front of the gene	this work
pEKEx3	Spec^R^; *E. coli*/*C. glutamicum* shuttle vector for regulated gene expression (P_*tac*_, *lacI*^q^, pBL1 *oriV_*Cg*_*)	Stansen et al., 2005
pEKEx3-*crtE2Y*	pEKEx3 derivative for IPTG-inducible expression of *crtE2Y* from *M. luteus*	Heider et al., 2014b
pEKEx3-*lbtABC*	pEKEx3 derivative for IPTG-inducible expression of *lbtABC* from *Dietzia* sp. CQ4	Heider et al., 2014b
pEPR1	Kan^R^; vector for transcriptional fusion analysis with the promoter-less *gfp*_uv_ reporter gene	Knoppova et al., 2007
pEPR1-P*crtE*	pEPR1 derivate with 5′UTR region of *crtE*	this work
pEPR1-PcrtE_M1^*^	pEPR1 derivate with 5′UTR region of *crtE* with mutation in motif 1 (TTAA to GGCC)	this work
pEPR1-PcrtE_M2^*^	pEPR1 derivate with 5′UTR region of *crtE* with mutation in motif 2 (AAATTT to CCCGGG)	this work
pEPR1-PcrtE_M12^*^	pEPR1 derivate with 5′UTR region of *crtE* with mutation in motif 1 (TTAA to GGCC) and 2 (AAATTT to CCCGGG)	this work
pEPR1-P*crtR*	pEPR1 derivate with 5′UTR region of *crtR*	this work
pEPR1-PcrtR_M1^*^	pEPR1 derivate with 5′UTR region of *crtR* with mutation in motif 1 (TTAA to GGCC)	this work
pEPR1-PcrtR_M2^*^	pEPR1 derivate with 5′UTR region of *crtR* with mutation in motif 2 (AAATTT to CCCGGG)	this work
pEPR1-PcrtR_M12^*^	pEPR1 derivate with 5′UTR region of *crtR* with mutation in motif 1 (TTAA to GGCC) and motif 2 (AAATTT to CCCGGG)	this work
pET16b	Amp^*R*^; overproduction of decahistidine tagged proteins in *E. coli* (pBR322 *oriV*_E.c._, *PT7, lacI*)	Novagen
pET16b-*crtR*	pET16b derivative for the purification of His-tagged CrtR of *C. glutamicum* from *E. coli* BL21(DE3)	this work
**OLIGONUCLEOTIDES**		
*crtR*-NdeI-fw	AAAA*CATATG*CTGAATATGCAGGAACC	
*crtR*-BamHI-rv	AAAA*GGATCC*CTACTCCGTGTTGAGCCATG	
SFT3	ATTCAGCATAGTAATCACCT	
SFT4	CATAAAAATAATGTGCCTAC	
SFT8	TCGAGTATCACACGGCCA	
SFT10	AACTCATGGGATACTATAAATTTC	
SFT12	AAAAATATTAACTCATGGGATACT	
SFT23	AAAAATATTAACTCATGGGATACTAT**CCCGGG**CTTGTAGGCACATTA	
SFT25	AAAAATA**GGCC**CTCATGGGATACTAT**CCCGGG**CTTGTAGGCACATTA	
SFT26	AAAAATA**GGCC**CTCATGGGATACTATAAATTTCTTGTAGGCACATTA	
SFT27	CACCAATACTACGTTCCACAT	
SFT28	GCCTACAAGAAATTTATAGTAT	
SFT29	CCCATGAGTTAATATTTTTAAAAAT	
SFT30	AAAAATAAACTTTATCTGACTTTGT	
cg2228_fw	CTCAGGCATGATGATGTCAGGC	
cg2228_rv	GTTCGCTACGTCCGAGTGATCACC	
*crtR*_A	GCAGGTCGACTCTAGAGGATCCCCGCGCGAAGATTTGATGGG	
*crtR*_B	CCCATCCACCCCGGGTAAACATTCCTGCATATTCAGCATAGTAATC	
*crtR*_C	TGTTTACCCGGGGTGGATGGGTCCCTTAATAATGCACCATGGC	
*crtR*_D	CCAGTGAATTCGAGCTCGGTACCCCTTGTCACCACAGCACTACT	
*crtR*_E	GCGCGAAGATTTGATGGG	
*crtR*_F	ACTTGTCACCACAGCACTAC	
*crtR*-fw	CTGCAGGTCGACTCTA**GAGGAAAGGAGGCCCTTCAG**ATGCTGAATATGCAGGAAC	
*crtR*-rv	CGGTACCCGGGGATCCTACTCCGTGTTGAGCCATG	
P*crtE*-pEPR-fw	CATGATCATCTAGAGAATTCGATTCAGCATAGTAATCACCT	
P*crtE*-pEPR-rv	GCCCGCTGAACTTGGATCTCGAGTATCACACGGCCA	
P*crtR*-pEPR-fw	CATGATCATCTAGAGAATTCGTCGAGTATCACACGGCCA	
P*crtR*-pEPR-rv	GCCCGCTGAACTTGGATCATTCAGCATAGTAATCACCT	
SDM-fw-nat	TAGTATCCCATGAGTTAATATTTTTAAA	
SDM-fw-mut	TAGTATCCCATGAG**GGCC**TATTTTTAAA	
SDM-rv-nat	ATGGGATACTATAAATTTCTTGTAGGCA	
SDM-rv-mut	ATGGGATACTAT**CCCGGG**CTTGTAGGCA	

*Sequence in bold: artificial ribosome binding site/mutated sequence*.

*Sequence underlined: linker sequence for hybridization*.

*Sequence in italic: restriction site*.

* *Sequence motif mutated by site-directed mutagenesis*.

### Recombinant DNA work

Plasmids were constructed in *E. coli* DH5α from PCR-generated fragments (KOD, Novagen, Darmstadt, Germany) and isolated with the NucleoSpin kit (MACHEREY-NAGEL GmbH & Co. KG, Düren, Germany). Oligonucleotides used in this study were obtained from Eurofins MWG Operon (Ebersberg, Germany) and Metabion (Planegg/Steinkirchen, Germany) and are listed in Table 1. Plasmid construction was executed by standard PCR, restriction and ligation reactions as described previously (Sambrook and Russell, 2001) as well as Gibson assembly (Gibson et al., 2009). The RbCl method was used for transformation of *E. coli* (Hanahan, 1983) and *C. glutamicum* was transformed via electroporation at 2.5 kV, 200 Ω, and 25 μF (Eggeling and Reyes, 2005). The correctness of the cloned DNA fragments was verified by sequencing.

### Homologous overexpression of genes from *C. glutamicum*

Plasmids for inducible gene expression were constructed on the basis of pEKEx3 (Stansen et al., 2005) or pVWEx1 (Peters-Wendisch et al., 2001). The gene *crtR* (cg0725) was amplified from genomic DNA of *C. glutamicum* WT, which was prepared as described (Eikmanns et al., 1995). Amplification was carried out by PCR using the respective *crtR*-fw/rv primers (Table 1).

### Deletion of *crtR* in *C. glutamicum* strains

Deletion of the gene *crtR* (cg0725) was carried out using the suicide vector pK19*mobsacB* (Schäfer et al., 1994). The construction of pK19*mobsacB*-*crtR* has been described (Henke et al., 2016). The genomic regions flanking the gene to be deleted by homologous recombination were amplified from genomic DNA of *C. glutamicum* WT using the primer pairs *crtR*_A/B and *crtR*_C/D (Table 1). The PCR products were purified and assembled and simultaneously cloned into *Sma*I restricted pK19*mobsacB* by Gibson assembly, which resulted in the deletion vector pK19*mobsacB*-*crtR* (Table 1). Targeted gene deletion proceeds via two-step homologous recombination as described previously using the before mentioned deletion vector (Eggeling and Bott, 2005). The first recombination event, the integration of the vector into one of the gene flanking regions, was selected via kanamycin resistance. Integration of the deletion vector into the genome triggers sucrose sensitivity due to the expression of *sacB*, encoding a levansucrase. In a second recombination event the deletion vector is excised and clones can be selected upon sucrose-resistance. By PCR analysis of three selected mutants using the primer pair *crtR*_E/F, deletion of the respective gene was verified (Table 1). The PCR products were then sequenced and in-frame deletion of *crtR* was confirmed.

### Extraction of carotenoids from bacterial cell cultures

The extraction of carotenoids from *C. glutamicum* was performed as described previously (Heider et al., 2014b) using 0.8 or 1 ml aliquots of the cell cultures. The pigments were extracted from the cell pellets with a methanol:acetone mixture (7:3) at 60°C for 30 min with thorough vortexing every 10 min. When necessary, extraction was repeated to remove all visible colors from the cell pellet. Subsequently, extraction mixtures were centrifuged for 5 min at 13,000 × g and the clear supernatant was used for analysis.

### Analysis of carotenoids

The carotenoid content of cell extracts was determined through absorbance at 470 nm by high performance liquid chromatography (HPLC) analysis as described previously (Heider et al., 2014b). HPLC analyses were performed on an Agilent 1200 series HPLC system (Agilent Technologies GmbH & Co. KG, Böblingen, Germany), including a diode array detector (DAD) for UV/visible (Vis) spectrum recording for detection. Separation of the carotenoids was accomplished by application of a column system (all columns from CS Chromatographie Service GmbH, Langerwehe, Germany) consisting of a pre-column (10 × 4 mm MultoHigh 100 RP18-5) and a main column (ProntoSIL 200-5 C30, 250 × 4 mm). Alternatively, 50 μL of the sample was separated with a column system consisting of a precolumn (LiChrospher 100 RP18 EC-5, 40 × 4 mm) and a main column (LiChrospher 100 RP18 EC-5, 125 × 4 mm) with methanol (A) and methanol/water (9:1) (B) as mobile phase. The following gradient was used at a flow rate of 1.5 mL/min; 0 min B: 0%, 10 min B: 100%, 32.5 min B: 100%. Quantification of lycopene and β-carotene was performed running a standard curve with HPLC grade standards (Sigma-Aldrich, Taufkirchen, Germany). Due to the lack of appropriate standards for decaprenoxanthin, sarcinaxanthin and C.p. 450 the quantification was calculated based on a β-carotene standard and reported as β-carotene equivalents (*y* = 79.7x; *R*^2^ = 0.96). The standards were dissolved in chloroform according to its solubility and diluted in methanol:acetone (7:3) for generating a standard curve.

### Transcriptome analysis

For transcriptome analysis triplicate cultures were grown in LB or CgXII medium. At an optical density of OD_600_ between 4 and 5 cells were harvested and total RNA was isolated with the RNeasy Mini Kit (Qiagen, Hilden, Germany) following the manufacturer's protocol. DNA was degraded in two reactions, in solution as well as on-column, using the RNase-Free DNase set (Qiagen, Hilden, Germany). Fluorescently labeled cDNA from 20 μg of total RNA was generated by indirect labeling as described by Hüser et al. (2003). cDNA probes labeled with Cy3 and Cy5 were combined for hybridization. Hybridization and scanning was performed as described earlier (Hüser et al., 2003). Signal-background segmentation, spot finding and intensity quantification were executed by ImaGene 6.0 software (BioDiscovery, Hawthorne, United States). Normalization (lowess) and *t*-test statistics were accomplished with EMMA 2.2, a software platform for consistent storage and efficient analysis of microarray data (Dondrup et al., 2003, 2009). Genes were regarded as differentially expressed when transcript abundance changed 2-fold or more (*M* ≥ 1 or *M* ≤ −1) with a statistical significance of *p* ≤ 0.05 in a student's *t*-test was determined.

### *In vivo* measurement of promoter activities of *crtR* and *crtE*

*In vivo* expression levels of *crtR* and *crtE* were assayed by transcriptional fusion of the intergenic region of *crtR* and *crtE* to the promoter-less GFP_*UV*_ gene using the pEPR1 plasmid system (Knoppova et al., 2007). Therefore, the corresponding promoter sequences were amplified from genomic DNA of *C. glutamicum* WT with primer pairs P*crtE*-pEPR-fw/P*crtE*-pEPR-rv and P*crtR*-pEPR-fw/P*crtR*-pEPR-rv, respectively and cloned into the *BamH*I restricted pEPR1 vector upstream of the promoter-less *gfp*_*UV*_ gene. Site-directed mutagenesis of putative motifs was performed on both constructed vectors using the primer pairs SDM-fw-nat/ SDM-rv-mut, SDM-fw-mut/ SDM-rv-nat and SDM-fw-mut/SDM-rv-mut. The GFP_UV_ shows characteristic emission at 509 nm with an excitation wavelength of 385 nm. Measurements of fluorescence were performed on a FACS Gallios™ (Beckmann Coulter GmbH, Krefeld, Germany) with 405 nm excitation from a blue solid-state laser. Forward-scatter characteristics (FSC) and side-scatter characteristics (SSC) were detected as small- and large-angle scatters of the 405 nm laser. GFP_*UV*_ fluorescence was detected using a 500/50 nm band-pass filter. *C. glutamicum* MB001Δ*crtR* and MB001 harboring the plasmids pEPR1-*PcrtR* and pEPR1-*PcrtE* or the plasmid with the mutated promoter sequences, were harvested in mid-exponential growth washed once in phosphate-buffered saline and the optical density was adjusted to OD_600_ <1. Cells harboring the empty vector pEPR1 were used to determine background fluorescence.

### Overproduction and purification of the transcriptional regulator CrtR

The vector pET16b-*crtR* was constructed using PCR-amplified *crtR* using oligonucleotides *crtR*-NdeI-fw and *crtR*-BamHI-rv and ligating it into pET16b restricted with *Bam*HI and *Nde*I. *E. coli* BL21(DE3) cells carrying the plasmid pET16b-*crtR* were grown at 37°C in 500 ml LB medium with 100 μg/ml ampicillin to an OD_600_ of 0.5 before adding IPTG for induction of the gene expression to a final concentration of 0.5 mM. After induction, cells were cultivated at 21°C for additional 5 h. Cells were harvested by centrifugation and stored at −20°C. For cell extract preparation, thawed cells were re-suspended in TNI buffer (20 mM Tris-HCl, pH 7.9, 300 mM NaCl, 5% (v/v) glycerol) with 5 mM imidazole containing a proteinase inhibitor cocktail tablet (Complete Mini, Roche, Basel, Switzerland). Cells were disrupted by ultrasonification using Hielscher UP200S2 (Teltow, Germany) with an amplitude of 60% and a pulsing cycle of 0.5 (power discharge 0.5 s, pause 0.5 s) for 2 min. After ultracentrifugation (1.5 h, 45,000 × g, 4°C) the supernatant was filtered through a 0.2 μm filter and purified by nickel affinity chromatography using nickel-activated nitrilotriacetic acid-agarose (NTA) (Novagen, San Diego, USA). TNI buffer containing 5 mM or 100 mM imidazole was used for sequentially washing of the column. The regulator protein was eluted with TNI buffer containing 400 mM imidazole and the fractions of highest protein concentrations were pooled. The elution buffer was exchanged against band shift (BS) buffer (50 mM Tris–HCl, 10% (v/v) glycerol, 50 mM KCl, 10 mM MgCl_2_, 0.5 mM EDTA, pH 7.5) using PD10 columns (GE Healthcare, Chalfont St. Giles, Great Britain). Protein concentrations were determined with the Bradford assay kit (Bio-Rad Laboratories, Hercules, Canada) using bovine serum albumin as reference. Protein purification was ascertained by 12% SDS-PAGE (polyacrylamide gel electrophoresis). The purified protein was applied for band shift assay without removing the N-terminal His-tag.

### Electrophoretic mobility shift assay (EMSA)

For verification of a physical Protein-DNA interaction between the regulator CrtR and the promoter region of *crtE* and *crtR*, band shift assay was performed according to Krause et al. (2012). His-tagged CrtR in varying molar excess was mixed with 90 ng of purified promoter-fragments of the target genes in band shift (BS) buffer (50 mM Tris–HCl, 10% (v/v) glycerol, 50 mM KCl, 10 mM MgCl_2_, 0.5 mM EDTA, pH 7.5) in a total volume of 20 μL. The 5′UTR of *crtE* was PCR-amplified with the oligonucleotides SFT8 and SFT3 (fragment A) listed in Table 1 and purified with NucleoSpin kit (MACHEREY-NAGEL GmbH & Co. KG, Düren, Germany). Truncated and mutated promoter fragments were amplified using oligonucleotide pairs SFT8/SFT10 (fragment B), SFT8/SFT12 (fragment C), SFT4/SFT27 (fragment D), SFT28/SFT27 (fragment E), SFT29/SFT27 (fragment F), SFT30/SFT27 (fragment G), SFT8/SFT23 (fragment H), SFT8/SFT26 (fragment I), SFT8/SFT25 (fragment K). A 78 bp-fragment of the upstream region of cg2228 was added in every sample as a negative control using oligonucleotides cg2228_fw and cg2228_rv. After 30 min of incubation at room temperature, gel shift samples were separated on a 6% DNA Retardation gel (Life Technologies GmbH, Darmstadt, Germany) at 100 V buffered in 44.5 mM Tris, 44.5 mM boric acid, and 1 mM EDTA at pH 8.3. Additionally, the binding affinity in presence of selected effectors was analyzed by incubation of the protein with the effector substance under buffered conditions for 15 min at room temperature prior to the addition of the promoter. Subsequently, the gel shift samples were separated on a 6% DNA Retardation gel (Life Technologies GmbH, Darmstadt, Germany) at 100 V buffered in 44.5 mM Tris, 44.5 mM boric acid, and 1 mM EDTA at pH 8.3. Staining of the DNA was achieved with ethidium bromide.

## Results

### Co-occurrence of the MarR-type regulator gene *crtR* with genes of carotenoid biosynthesis in completely sequenced actinobacterial genomes

In the genome of *C. glutamicum* the carotenogenic genes responsible for conversion of the building blocks IPP and DMAPP to decaprenoxanthin are organized in the *crt* operon (*crtE-cg0722-crtBIY*_*e*_*Y*_*f*_*Eb*) (Heider et al., 2012). The major GGPP synthase IdsA (Heider et al., 2014a) and a second phytoene synthase are encoded elsewhere on the chromosome (Heider et al., 2012). Divergent to the *crt* operon and 200 bp upstream of its first gene, *crtE*, one of nine MarR-type transcriptional regulator genes in the genome of *C. glutamicum* (Brune et al., 2005) is found. This gene cg0725 was named *crtR* based on the results described in this study. Previously, a transposon mutagenesis screen showed that insertion of a transposon into *crtR* enhanced carotenoid production (Krubasik et al., 2001a). A model of the protein secondary structure of the 195 aa containing *crtR* product revealed a pfam12802 domain with a centrally located winged HTH motif located from aa position 73 to 134 as is typical for MarR-type regulator proteins of *C. glutamicum* (Brune et al., 2005).

**Figure 1:**
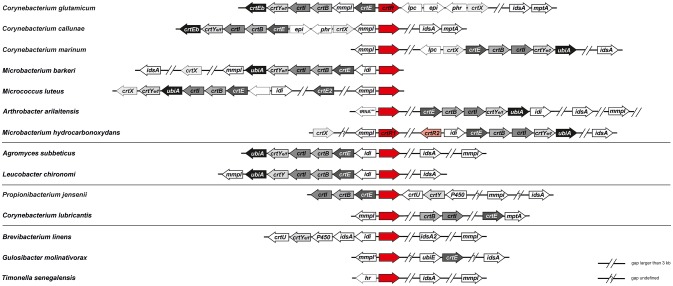
**Co-occurrence of homologs of *C. glutamicum crtR* with genes of carotenogenesis in various completely sequenced genomes**. The genetic organization of *crtR* homologs and genes relevant for carotenoid biosynthesis is depicted for representative genomes containing a *crtR* homologue and at least one gene of carotenogenesis (s. also Figures S1, S3). *crtR*, regulator of carotenoid biosynthesis and its homologs (in red); *crtE*/*idsA*, geranygeranyl diphosphate synthase; *crtB*, phytoene synthase; *crtI*, phytoene dehydratase; *crtY*_e/f_, C50 epsilon cyclase; *crtEb*, lycopene elongase, *crtX*, carotenoid glycosyl transferase, *mmpl*, putative RND drug exporter; *mmpl'*, pseudogene with homology to *mmpl*; *lpc*, putative lipocalin; *phr*, putative deoxyribopyrimidine photolyase; *epi*, putative NDP sugar epimerase; *idi*, isopentenyl diphosphate isomerase; *crtEb*/*ubiA*, prenyltransferases; P450, cytochrome P450 monooxygenase; *mptA*, α(1→6) mannopyranosyltransferase; *ubiE*, ubiquinone biosynthesis methyltransferase; *crtU*, β-carotene desaturase/isorenieratene synthase; *crtY*, lycopene cyclase (no significant homology to CrtY_e/f_ from *C. glutamicum*); hr, hemerythrin.

A sequence comparison by BLAST showed that *C. glutamicum* CrtR is distinct from MarR-type regulators of *Bacillus subtilis, Escherichia coli, Mycobacterium*, and *Streptomyces* species (data not shown), but showed highest similarities to MarR-type proteins of the high GC content family of Actinobacteria, primarily of species of the genera *Corynebacterium, Microbacterium, Leucobacter, Arthrobacter, Propionibacterium*, and *Gulosibacter* (Figure S1). A sequence alignment with the closest relatives of CrtR (CAF19334) from *C. vitaeruminis, C. efficiens, C. callunae, Leucobacter* sp. Ag1, *M. mangrovi, Brevibacterium* sp. VCM10, and *L. chironomi* shows high similarity especially in the central region of the proteins where the proposed HTH motif is located (Figure S2). Since *crtR* is localized in proximity to the *crt* operon in *C. glutamicum*, it was tested if this pattern is conserved among bacteria containing a CrtR ortholog. First, sequence comparisons of *C. glutamicum* CrtR to completely sequenced genomes identified bacteria possessing a CrtR homolog showing at least 25% amino acid identity and an *e*-value <10^−10^. Subsequently, the completely sequenced genomes of these bacteria were analysed for the presence of homologs (25% amino acid identity and *e*-value <10^−10^) of the *C. glutamicum* proteins encoded by *crtEb* (cg0717), *crtY*_*f*_ (cg0718), *crtY*_*e*_ (cg0719), *crtI* (cg0720), *crtB* (cg0721), cg0722, *crtE* (cg0723), *crtX* (cg0730), and *idsA* (cg2384). Although the low identity cut-off of 25% may have led to more false-positives than searches with higher identity cut-offs (Rost, 1999), this was compensated for by the high significance value of e^−10^ and the combinatory search for CrtR homologs and other carotenogenesis genes. If at least one homolog of the proteins in this list were encoded in a completely sequenced genome in addition to a homolog of *C. glutamicum* CrtR, the genome was considered further and the results depicted as heatmap (Figure S3). Opposite and in divergent orientation to the *crtR* homologous genes, individual genes or *crt* operons were found. For the most part, the individual genes or the first genes of the *crt* operons encoded GGPP synthase (*crtE*), IPP isomerase (*idi*), and putative multidrug efflux protein (*mmpl*), respectively (Figure 1).

With respect to the genes encoding homologs of *C. glutamicum* CrtR and genes involved in carotenogenesis four distinct patterns emerged (Figure 1). First, a group of bacteria possessing a full complement of carotenogenesis genes including genes for carotenoid elongase (*crtEb*), carotenoid cyclase (*crtY, crtY*_*e*_*/Y*_*f*_) and a carotenoid glycosyltransferase (*crtX*). This group comprised bacteria known to produce glycosylated, cyclic C50 carotenoids (*C. glutamicum, C. callunae, C. marinum, Micrococcus luteus)*, but also bacteria of which it is unknown which carotenoids they synthesize (*Arthrobacter arilaitensis, Microbacterium barkeri*, and *Microbacterium hydrocarbonoxydans*; Figure 1). A second group with *Agromyces subbeticus* and *Leucobacter chironomi* lacks *crtX* homologs likely synthesizing non-glucosylated carotenoids and a third group with *Propionibacterium jensenii* and *Corynebacterium lubricantis* lacks *crtX* and *crtY* homologs likely synthesizing noncyclic nonglycosylated carotenoids. Finally, a group with *Brevibacterium linens, Gulosibacter molinativorax* and *Timonella senegalensis* lacks *crtX, crtY* and *crtE/ubiA* homologs and these are likely unable to synthesize glycosylated or cyclic or elongated carotenoids. Taken together, homologs of *C. glutamicum* CrtR are conserved in various bacteria (mostly actinobacteria) that were shown to synthesize various carotenoids (cyclic and noncyclic, elongated or not, glycosylated or not) or do have the potential to synthesize these according to their gene repertoire. Moreover, the *crtR* homologs are found in diverse genomic locations, but mostly adjacent to *crt* operons or genes involved in carotenogenesis. These findings supported the hypothesis that CrtR homologs may have a conserved role in regulation of carotenogenesis.

### CrtR negatively regulates expression of the *crt* operon and of its own gene

To test if *C. glutamicum* CrtR plays a role in transcriptional regulation of genes of carotenogenesis a *crtR* deletion mutant was constructed and characterized with respect to global gene expression. Comparative transcriptome analyses of *C. glutamicum* MB001Δ*crtR* and *C. glutamicum* MB001 were performed to identify genes directly or indirectly regulated by CrtR. The transcriptomes of cells cultured in triplicates either in glucose minimal medium or LB complex medium revealed only eight differentially expressed genes (Table 2). All of the differentially expressed genes showed 8 to 69-fold increased mRNA levels and they belonged to the *crt* operon or, as in the case of cg0726 that codes for a putative secreted lipoprotein, are localized adjacent to it. Deletion of *crtR* led to considerably higher mRNA ratios for cg0717 and cg0719 during growth in LB than in minimal medium, however, this does not pertain to the other genes of the *crt* operon. Currently, it remains unclear if these differences may be caused by technical issues or have a biological cause, however, it has to be noted that RNAseq analysis revealed expression of cg0717 and cg0718 independent of the other *crt* genes, thus, sub-operons may exist (Pfeifer-Sancar et al., 2013). As expected from the operon structure all transcripts were decreased from the first to the last genes in the *crt* operon. Taken together, the *crt* operon of *C. glutamicum* is directly or indirectly regulated by CrtR.

**Table 2:** **Genes differentially expressed in *C. glutamicum* strains MB001 and MB001Δ*crtR* during growth in minimal medium with 100 mM glucose as well as in complex LB medium**.

			**mRNA ratio^a^ (MB001Δ*crtR*/MB001)**	
Gene ID	Gene name	Annotation	LB	CgXII
cg0717	*crtEb*	Lycopene elongase	34	13
cg0718	*crtYf*	C50 carotenoid epsilon cyclase	33	35
cg0719	*crtYe*	C50 carotenoid epsilon cyclase	69	24
cg0720	*crtI*	Phytoene dehydrogenase (desaturase)	36	32
cg0721	*crtB*	Phytoene synthase	55	45
cg0722	−	Putative multidrug efflux protein, resistance-nodulation-cell division (RND) superfamily	13	22
cg0723	*crtE*	GGPP synthase	33	40
cg0726	−	Putative secreted lipoprotein	8	10

a *Average ratio of medians. Only genes regulated more than 2-fold in at least one strain were considered*.

*Only results with a p-value (adjusted with the False Discovery Rate approach) of ≤ 0.05 were considered to be significant. Three biological replicates were performed*.

The pEPR1-promoter probe vector system for transcriptional fusion analysis (Knoppova et al., 2007) was used to determine if CrtR regulates transcription of its own gene and of the *crt* operon *in vivo*. The intergenic region between *crtR* and *crtE*, the first gene of the *crt* operon, was cloned upstream of the promoter-less *gfp*_*UV*_ gene in both orientations (named P*crtE* and P*crtR*, respectively). Reporter gene expression in *C. glutamicum* MB001(pEPR1-P*crtE*), MB001(pEPR1-P*crtR*), MB001Δ*crtR*(pEPR1-P*crtR*), and MB001Δ*crtR*(pEPR1-P*crtE*) was determined by fluorescence cell scanning in LB medium in the early exponential growth phase (Table 3). About 2-fold higher expression of the P*crtR* fusion in the absence of CrtR revealed that CrtR is subject to negative autoregulation (Table 3). Expression of the P*crtE* fusion was about 5-fold higher when *crtR* was deleted indicating that CrtR represses transcription of the *crt* operon (Table 3).

**Table 3:** **Effect of *crtR* deletion on *crtE* and *crtR* promoter activity**.

	**pEPR1**	**Native**	M1^*^	M2^*^	M1^*^2^*^
**pEPR1-P*****crtE***					
MB001	0.21 ± 0.01	0.47 ± 0.02	3.89 ± 0.17	0.26 ± 0.01	0.79 ± 0.03
MB001Δ*crtR*	0.20 ± 0.01	2.64 ± 0.09	4.12 ± 0.01	0.57 ± 0.01	0.82 ± 0.02
**pEPR1-P*****crtR***					
MB001	0.21 ± 0.01	0.63 ± 0.01	0.48 ± 0.01	0.63 ± 0.02	0.45 ± 0.01
MB001Δ*crtR*	0.20 ± 0.01	1.10 ± 0.01	0.46 ± 0.03	1.43 ± 0.01	0.44 ± 0.02

**Figure 2:**
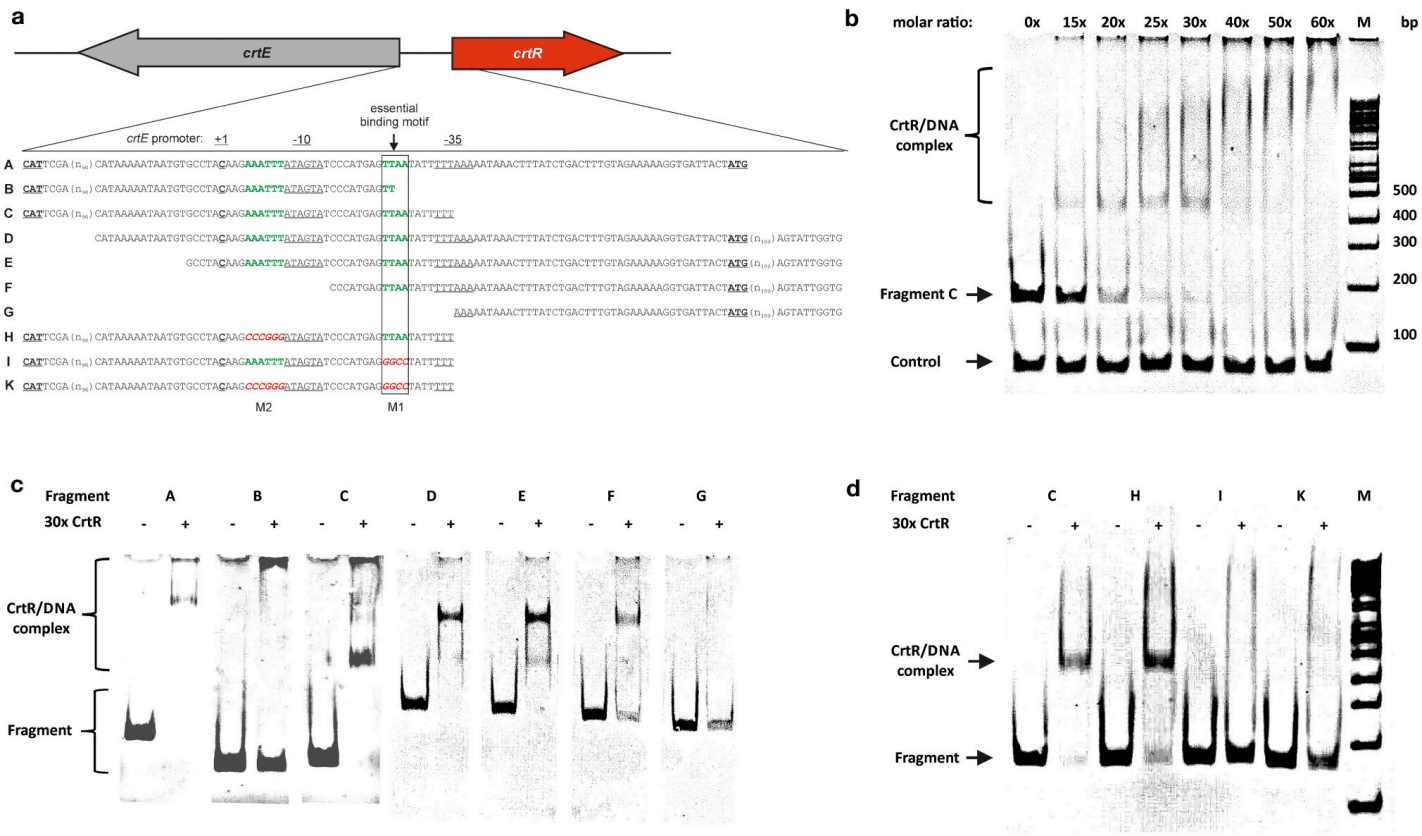
**DNA-binding studies of purified CrtR_His6_ protein with the 5′UTR of *crtE***. Promoter region of *crtE*/*crtR* and fragments used for electromobility shift assay (EMSA) **(a)**. In green putative binding motifs (M1 and M2) are depicted and in red the respective mutations. The transcriptional start site of the *crt*-operon is depicted as C in bold (Heider et al., 2012). Fragment A represents the full length intergenic region of *crtE* and *crtR* including both translational starts (in bold letters). Fragment B,C,H,I, and K are shortened from the 3′ end, possessing either the original sequence (B, C) or mutations (H, I, K). Fragments D, E, F, and G were ‘successively shortened from the 5′ end and’ constructed with an extra 190 bp downstream of the *crtR* translational start codon. **(b)** EMSA with fragment C and different molar rations of purified CrtR_His6_ from 0 (no protein added), 15, 20, 25, 30, 40, 50, and 60-fold molar excess. As a control the 5′UTR of cg2228 was used. **(c)** EMSA with 3′ (B, C) and 5′ (D, E, F, G) truncated fragments of the *crtE-crtR* intergenic region as depicted in a) and 30-fold molar excess of purified CrtR_His6_. **(d)** EMSA with 3′ truncated fragment C and mutated fragments (H, I, K) and 30-fold molar excess of purified CrtR_His6_.

GFP_UV_ fluorescence medians of C. glutamicum MB001 strains with and without a genomic copy of crtR harboring either pEPR1-PcrtR or pEPR1-PcrtE or their mutated derivatives [M1^*^: motif 1 (TTAA → GGCC), M2^*^: motif 2 (AAATTT → CCCGGG) as in Figure 2] when grown on LB complex. Cells were harvested in exponential growth phase. GFP_UV_ fluorescence was detemined by FACS analysis. Mean values and standard deviations of three replicates are given.

### Gel mobility shift and transcriptional fusion analyses revealed that CrtR binds to the intergenic region between *crtR* and *crtE*

As CrtR was shown to negatively regulate transcription of the *crt* operon and of its own gene, gel mobility shift assays were performed to determine if CrtR directly interacts with the intergenic region between *crtR* and *crtE*. Therefore, CrtR protein was produced in recombinant *E. coli* BL21 (DE3) as N-terminal His-tagged protein and purified to apparent homogeneity by affinity chromatography (Figure S4). Gel mobility shift analysis revealed that His-tagged CrtR bound to the intergenic region between *crtR* and *crtE* (fragment A; Figures 2a,c), whereas BSA as a control did not bind to this DNA fragment (data not shown). At a 30 to 40-fold molar excess of CrtR a complete shift of the promoter fragment C was observed, whereas a negative control fragment comprising the sequence upstream of the unrelated gene cg2228 was not bound by CrtR (Figure 2b). To localize the CrtR binding site, truncated fragments of the intergenic region were used for further gel shift assays. In line with the observation that MarR-type regulators typically bind to palindromic sites of 16–20 bp which often overlap the −10 and −35 regions for steric inhibition of RNA polymerase (Wilkinson and Grove, 2006), two inverted repeats were found in the intergenic region between *crtR* and *crtE* (Figure 2a). CrtR bound to fragments A, C, D, E, and F that contained a TTAA sequence motif 25–28 bases upstream of the transcription start site of *crtE* (Figures 2c,d). Fragments B and G that lack the complete TTAA motif were not bound by CrtR (Figures 2c,d) indicating that this sequence motif might be essential for CrtR binding. Thus, this motif and a similar motif (AAATTT overlapping with the −10 hexamer of the *crtE* promoter; Figure 2a) were mutated. Mutating the latter motif (fragment H) did not influence binding of CrtR, whereas CrtR did not bind to fragments with a mutated TTAA motif (fragments I and K; Figure 2d). Thus, the TTAA motif was shown to be required for binding of CrtR *in vitro*.

In order to determine the role of the TTAA motif for regulation by CrtR *in vivo*, transcriptional fusions of the promoters P*crtR* and P*crtE* containing mutations in the TTAA and/or the AAATTT motifs were constructed. Mutation of the AAATTT motif changed the −10 hexamer of the P*crtE* promoter from TATAAA to TATGGG (Figure 2a). Consequently, only very low reporter gene activities were measured (Table 3) and thus, the relevance of the AAATTT motif for regulation of the P*crtE* promoter could not be determined. Reporter gene activities of a transcriptional fusion of P*crtR* containing the mutated AAATTT motif were comparable to those obtained with the non-mutated P*crtR* promoter and revealed that this motif is dispensable for negative autoregulation by CrtR (Table 3). Mutation of the TTAA motif led to very low reporter gene activity with P*crtR*, which precluded deducing its involvement in *crtR* autoregulation. By contrast, mutation of the TTAA motif led to high reporter gene activities of P*crtE* regardless of the absence or presence of CrtR (Table 3) which supports the notion that this motif is required for function of CrtR as transcriptional repressor of the *crt* operon *in vivo*.

### Isoprenoid pyrophosphates prevent binding of CrtR to its DNA target

**Figure 3:**
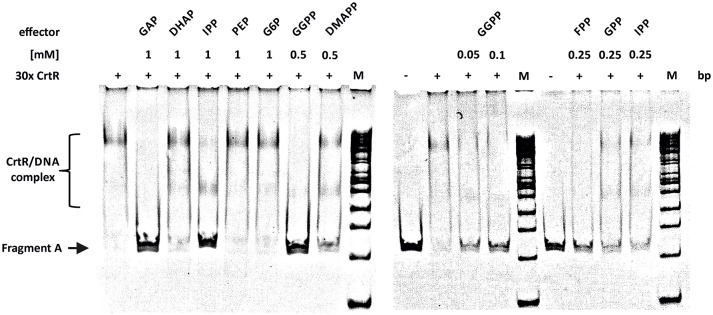
**Electromobility shift assay of purified CrtR_His6_ protein in the presence of different low molecular weight molecules**. Analysis of phosphorylated intermediates from the carotenoid biosynthesis pathway as potential effector molecules on the interaction of CrtR_His6_ with the full length intergenic region of *crtE* and *crtR*. The purified CrtR_His6_ was used in 30 molar excess and the effector molecules were used in depicted concentrations.

Regulation of carotenogenesis in response to low-molecular-weight compounds has not yet been described. However, since CrtR belongs to the MarR family of transcriptional regulators and representatives of this family show regulation in response to low-molecular-weight compounds, such as hydrogen peroxide in the case of RosR of *C. glutamicum* (Bussmann et al., 2010), low-molecular-weight compounds were tested as effectors of CrtR in gel mobility shift assays. Intermediates of carotenogenesis (isoprenoid pyrophosphates), precursors of the MEP pathway (GAP and pyruvate), but also phosphorylated intermediates of glycolysis (DHAP, phosphoenolpyruvate and D-glucosamine 6-phosphate), organic acids (acetate, propionate) and compounds affecting other MarR-type regulators [vitamin B12, salicylic acid, urea, protocatechuic acid ‘(data not shown)’] were tested as effectors of CrtR with concentrations in the mM range. Neither DHAP (Figure 3) nor pyruvate (data not shown) had an effect on CrtR binding to its DNA target. In the contrary, in the presence of GAP CrtR binding was prevented (Figure 3). In addition, the isoprenoid pyrophosphates IPP, DMAPP, geranyl pyrophosphate (GPP), farnesyl pyrophosphate (FPP) and GGPP perturbed binding of His-tagged CrtR to the intergenic region between *crtR* and *crtE* (Figure 3). Among these isoprenoid pyrophosphates, GGPP was effective at the lowest concentration (50 μM), whereas FPP, GPP, IPP and DMAPP exerted similar effects on CrtR binding *in vitro* at a 5-fold higher concentration (Figure 3). Thus, GGPP, shorter isoprenoid pyrophosphates and the MEP-pathway precursor GAP interfere with the *in vitro* binding of CrtR to the intergenic region between *crtR* and *crtE* and the strength of interference follows the order GGPP >> FPP, GPP, IPP, DMAPP >> GAP (Figure S5).

### Deletion of *crtR* is a metabolic engineering strategy to increase production of native and non-native carotenoids

Since CrtR represses the *crt* operon the effect of deletion and overexpression of *crtR* on decaprenoxanthin accumulation in cells of *C. glutamicum* wild type and the phage-cured strain MB001 was determined. While growth was not significantly affected by the deletion or overexpression of *crtR*, neither in complex medium (data not shown) nor in minimal medium, the *crtR* deletion mutant showed more intense yellow pigmentation, while pigmentation of the overexpressing strain was slightly decreased (Figure S6). HPLC analyses of extracts from cells grown in CGXII medium revealed that the *crtR* deletion mutants accumulated decaprenoxanthin to 15 to 30-fold higher levels than the corresponding parental strains (Table 4). By contrast, as consequence of plasmid-borne overexpression of *crtR* decaprenoxanthin levels were significantly decreased (Table 4).

**Table 4:** **Decaprenoxanthin accumulation and growth rates in the presence and absence of the *crtR* gene and its plasmid-borne overexpression in *C. glutamicum* WT and MB001 strains**.

**Strain**	**Decaprenoxanthin concentration [mg/g CDW]**
WT	0.24 ± 0.03
WT Δ*crtR*	3.57 ± 0.12
WT (pVWEx1)	0.29 ± 0.01
WT (pVWEx1-*crtR*)	0.05 ± 0.01
MB001Δ*crtR* (pVWEx1)	4.07 ± 0.69
MB001Δ*crtR* (pVWEx1-*crtR*)	0.04 ± 0.01
MB001(pVWEx1)	0.12 ± 0.01

*C50 carotenoid quantities are given as β-carotene equivalents. Cells were grown in glucose CGXII minimal medium for 24 h induced by 1 mM IPTG. Mean values and standard deviations of three replicates are given*.

**Figure 4:**
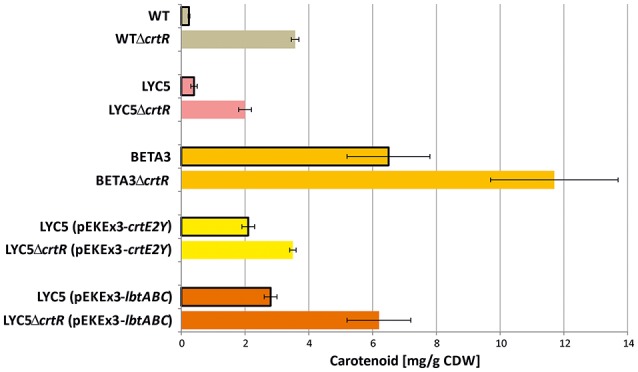
**Application of regulator engineering on C40 and C50 carotenoid producing *C. glutamicum* strains**. C50 carotenoid quantities are given as β-carotene equivalents. Cells were grown in glucose CGXII minimal medium for 24 h induced by 1 mM IPTG. Mean values and standard deviations of three biological triplicates are given [except LYC5Δ*crtR* (pEKEx3-crtE2Y)]. Data from WT and WTΔ*crtR* are taken from Table 3. *p* < 0.05 between control and Δ*crtR* strains in students *t*-test (two-sided, unpaired). Gray, decaprenoxanthin; pink; lycopene; light orange, β-carotene; yellow, sarcinaxanthin; red, C.p.450.

Since lycopene is a central intermediate of C40 and C50 carotenoid biosynthesis, the effect of deletion of *crtR* was elucidated in a metabolically engineered lycopene producing LYC5. Lycopene concentration increased about 5-fold (Figure 4). To test if this type of regulatory engineering can also be applied to the production of non-native carotenoids, *crtR* was deleted in the β-carotene producing strain BETA3, the sarcinaxanthin producing strain LYC5 (pEKEx3-*crtE2Y*), and the C.p.450 (2,2′-bis-(4-hydroxy-3-methybut-2-enyl)-β,β-carotene) producing strain LYC5 (pEKEx3-*lbtABC*). Indeed, all strains lacking CrtR produced 1.5 to 2 fold more carotenoid than the parental strains (Figure 4). Thus, deletion of *crtR* is a general strategy to boost production of native and non-native C40 and C50 carotenoids by *C. glutamicum*.

## Discussion

In this study, the MarR-type transcriptional regulator CrtR was shown to repress the *crt* operon, its own gene and likely also the adjacent gene cg0726. GGPP and to lesser extent the isoprenoid pyrophosphates FPP, GPP, IPP, and DMAPP as well as the MEP pathway precursor GAP interfered with CrtR binding *in vitro* to a TTAA containing sequence in the intergenic region between *crtR* and the *crt* operon overlapping with the *crt* operon promoter.

CrtR is the first MarR-type regulator repressing carotenogenic genes in bacteria that is characterized to some detail. Commonly, RNA polymerase σ factors and transcriptional regulators of a different protein family (MerR-type, but not MarR-type) are involved in repression of carotenogenic operons in non-phototrophic bacteria. Unlike CrtR, these MerR-type regulators bind vitamin B12 as corepressor. By contrast, CrtR lacks a vitamin B12 binding domain (Figure S2) but EMSA experiments revealed that its capacity to bind target DNA is influenced by isoprenoid pyrophosphates (Figure 3). The MerR-type regulators CarH, CarA and LitR involved in regulation of carotenogenic operons in non-phototrophic bacteria have been characterized (Gorham et al., 1996; Whitworth and Hodgson, 2001; Fontes et al., 2003; Takano et al., 2005, 2006). In the complex regulation of carotenogenesis in *M. xanthus*, two MerR-type regulators (CarH and CarA), an anti-repressor (CarS), a RNA polymerase extracytoplasmic function (ECF)-σ factor (CarQ), anti-σ factor (CarR) and anti-anti-σ factor (CarF) are involved. The carotenogenic *carB* operon in *M. xanthus* is repressed in the dark by CarA and/or CarH, the latter of which requires binding of vitamin B12 as corepressor. Light induction is mediated by the SH3 domain-containing anti-repressor CarS, which binds to the DNA binding domains of CarA (Leon et al., 2010) and CarH/B12-complex and therefore counteracts repression of the carotenogenic *carB* operon (Galbis-Martinez et al., 2012). CarS itself is only synthesized in the light, since the *carQRS* operon is transcribed in the light by CarQ. The ECF-σ factor CarQ is only available when its anti-σ factor CarR is inactivated by anti-anti-σ factor CarF in response to light induced formation of singlet oxygen by heme precursor protoporphyrin IX interacting with molecular oxygen (Galbis-Martinez et al., 2012).

Light induction may also be regulated directly by the MerR-type regulators functioning as vitamin B12-dependent photoreceptors (Jost et al., 2015). Carotenoid production in Gram-negative *T. thermophilus* is characterized by *litR* and *crtB*, the first gene of the *crt* operon, both being repressed in the dark by the LitR with 5′-deoxyadenosylcobalamin (AdoB12) bound as corepressor (Jost et al., 2015; Takano, 2016). In the light vitamin B12, the chromophore of the tetrameric AdoB12-LitR-complex is photolysed to hydroxycobalamin, which causes a large-scale conformational change of the regulator. This leads to the loss of operator binding and therefore derepression of its own gene and the subsequent cAMP-CRP family activator gene *ldrP* (Ortiz-Guerrero et al., 2011). This LdrP protein activates transcription of the *crt* operon, DNA photolyase gene *phrB*, and some further genes (Takano et al., 2014). LitR of *S. coelicolor* also functions as vitamin B12-dependent photoreceptor and its own gene and the ECF-σ factor gene *litS* are derepressed upon illumination. As consequence, RNA polymerase holoenzyme containing σ^LitS^ transcribes the *crt* operons (Takano, 2016). *B. megaterium* possesses a LitR homologue as vitamin B12-dependent photoreceptor. The *crt* operon is relieved from repression by LitR in the light, however, a dedicated σ factor as in *S. coelicolor* is not involved since transcription occurs by RNA polymerase holoenzyme containing σ^A^ (Takano et al., 2015a). In *C. glutamicum*, however, the MarR-type transcriptional regulator CrtR has been shown here to repress the carotenoid biosynthesis operon. Based on the *in vitro* analysis it is hypothesized that isoprenoid pyrophosphates act as inducers. Hitherto, neither MarR-type regulators nor low-molecular-weight ligands, such as intermediates of the MEP-pathway or carotenogenesis have been reported to be involved in transcriptional regulation of microbial or plant carotenoid biosynthesis.

MarR-type transcriptional regulators are categorized according to their physiological function acting as regulators of (i) the stress response, (ii) virulence factors or (iii) aromatic catabolism (Wilkinson and Grove, 2006). Typically, MarR-type regulators are sensing environmental changes and some of them regulate drug efflux pump gene expression (Grove, 2013). *C. glutamicum* is equipped with nine MarR-type regulators (Brune et al., 2005) of which only the hydrogen peroxide-responsive activator of the nitrate/nitrite transporter and the dissimilatory nitrate reductase complex genes RosR (Bussmann et al., 2010), the repressor of the malic enzyme gene MalR (Krause et al., 2012), and PhdR, the repressor of the genes required for β-oxidation of phenylpropanoids (Alekshun et al., 2001), have been analyzed to date. MarR proteins are mostly encoded either as part of the regulated gene cluster or their genes are localized adjacent to and in divergent transcriptional organization to the regulated genes (Wilkinson and Grove, 2006). The latter case applies for CrtR, MalR, RosR, and PhdR of *C. glutamicum* WT. Although N-terminal HTH domains are most common for negative transcriptional regulators (Perez-Rueda et al., 1998), CrtR possesses a central HTH domain preceded by 72 amino acids (corresponding to aa 73–134). This is also true for MalR, RosR and PhdR, however, their HTH domains are only preceded by 36 to 47 N-terminal amino acids and these proteins are about 40 amino acids shorter than CrtR.

The regulon of CrtR is small, comprising only the *crt* operon, its own gene and possibly cg0726. CrtR may have only one DNA binding site on the *C. glutamicum* genome which is located between *crtR* and the *crt* operon and comprises a TTAA motif required for CrtR binding (Figure 2). This motif is located 25–28 bp upstream of the transcription start site of *crtE*, which was identified 114 nucleotides upstream of the start codon (Heider et al., 2012). This motif overlaps the −35 and/or −10 promoter elements of the *crt* operon, which for other MarR-type regulators was shown to indicate that repression is achieved by steric inhibition of RNA polymerase binding to the promoter (D'Souza et al., 1997). Although short, this motif is palindromic as generally observed for most MarR-type transcriptional regulators (Kelly et al., 1970; Ronnekleiv, 1995). When using promoter fusions of P*crtE* and P*crtR*, a more intense yellow pigmentation in comparison to the empty vector carrying control strains was observed (data not shown), which likely is due to a titrating effect by the CrtR binding site present on the medium copy promoter-probe vectors (Korshunov and Imlay, 2006). The more intense yellow pigmentation was not observed when the CrtR binding site in the promoter probe vectors was mutated. Deletion of *crtR* derepressed the *crt* operon by about 5-fold, but derepressed its own gene by about 2-fold, which might indicate that P*crtE* is stronger than P*crtR*. The −10 hexamer of P*crtE* does not deviate from the consensus sequence TANNNT (Pfeifer-Sancar et al., 2013), while the −35 promoter hexamer TTTAAA consensus (TTGNCA) hexamer is less conserved. However, transcription of *crtR* and its autoregulation are less well understood since promoter motifs for *crtR* cannot easily be found (Figure 2a), although it has been reported that *crtR* is transcribed as a leaderless transcript (Pfeifer-Sancar et al., 2013). A surprisingly high fraction of transcripts in *C. glutamicum* (33%) lack an 5′ untranslated region (Pfeifer-Sancar et al., 2013) and it remains to be studied if leaderless transcripts are translated less efficient than transcripts containing leaders (Moll et al., 2004) and if they are resistant to inhibition by kasugamycin as is the case for *E. coli* (Lange et al., 2016).

MarR-type regulators are often involved in stress responses and conformational changes due to binding of an effector molecule impair their binding to their specific DNA targets, as it was described e.g., for MarR in its salicylated form (Alekshun et al., 2001). The MarR-type regulator of oxidative stress response RosR of *C. glutamicum* showed reduced binding to its target DNA when exposed to H_2_O_2_. Furthermore, H_2_O_2_ sensitivity was increased in the absence of the *rosR* adjacent gene cg1322, which is proposed to encode a secreted protein likely binding an octaprenyl pyrophosphate molecule. Since menaquinone contains an octaprenyl side chain and has an effect on the flux of superoxide to the periplasm of *E. coli* (Korshunov and Imlay, 2006), a correlation between oxidative stress and polyisoprenoid structures in the cytoplasmic membrane of *C. glutamicum* has been postulated (Bussmann et al., 2010). In this study, an influence of typical MarR effectors, such as acetate, propionate, salicylic acid, urea or protocatechuic acid could not be observed (data not shown). Vitamin B12 involved in LitR-mediated control of carotenogenesis in *S. coelicolor* (Takano et al., 2005), did not affect binding of CrtR to *PcrtE*. However, we cannot exclude that the CrtR preparations from *E. coli* are free from low molecular weight compounds.

Here, we have described that isoprenoid pyrophosphates interfered with binding of CrtR to its cognate DNA target. Consistently, many members of the MarR family bind similar, mostly anionic lipophilic or even phenolic effector molecules (Wilkinson and Grove, 2005). The interfering effect of isoprenoid pyrophosphates was specific as vitamin B12, phosphoenolpyruvate, D-glucosamine 6-phosphate, acetate, propionate, salicylic acid, urea or protocatechuic acid had no significant influence on CrtR binding (data not shown). Based on these *in vitro* results, it is tempting to speculate that CrtR may be used as a potential biosensor of IPP, DMAPP, GPP, FPP, and GGPP for strain engineering and optimizing terpenoid production by controlling gene expression *on demand*. This kind of strain development has been shown to work efficiently using photocaged-IPTG as applied to valencene production by *C. glutamicum* (Binder et al., 2016) or using riboswitches as applied to lysine production by *C. glutamicum* (Zhou and Zeng, 2015a,b).

CrtR is not only found in corynebacteria, but conserved in actinobacteria that, based on their genome content, have the potential to synthesize various carotenoids (Figure 1). It has to be noted that the genomic organization of *crtR* in most of these bacteria is linked to genes involved in carotenogenesis. Thus, it is not unlikely that CrtR plays a comparable role in transcriptional regulation of carotenogenic genes in these bacteria.

## Author contributions

All authors planned and designed the experiments. NAH, SAEH, and SH performed the experiments and analyzed the data. PPW and VFW analyzed data. NAH, SAEH, and PPW drafted the manuscript. VFW and PPW coordinated the study and finalized the manuscript. All authors read and approved the final manuscript.

## Funding

We acknowledge support for the Article Processing Charge by the Deutsche Forschungsgemeinschaft and the Open Access Publication Fund of Bielefeld University.

### Conflict of interest statement

The authors declare that the research was conducted in the absence of any commercial or financial relationships that could be construed as a potential conflict of interest. The reviewer BL and handling Editor declared their shared affiliation, and the handling Editor states that the process nevertheless met the standards of a fair and objective review.
