# Supplementary material for: Isoprenoid Pyrophosphate-Dependent Transcriptional Regulation of Carotenogenesis in Corynebacterium glutamicum
Source: Front Microbiol. 2017 Apr 24;8:633. doi: 10.3389/fmicb.2017.00633 (PMC5401885; doi:10.3389/fmicb.2017.00633)
Supplement: Supplementary file 1 [file DataSheet1.PDF]

Supplementary Material

Isoprenoid pyrophosphate dependent transcriptional regulation of carotenogenesis in *Corynebacterium glutamicum*

Nadja A. Henke, Sabine A. E. Heider#, Silvin Hannibal, Volker F. Wendisch, Petra Peters-Wendisch*

[1] Genetics of Prokaryotes, Faculty of Biology, Center for Biotechnology (CeBiTec), Bielefeld University, Bielefeld, Germany

[#] Current Address: GSK Vaccines S.r.l., Siena, Italy

*** Correspondence:**
Petra Peters-Wendisch
petra.peters-wendisch@uni-bielefeld.de

Supplementary Figures

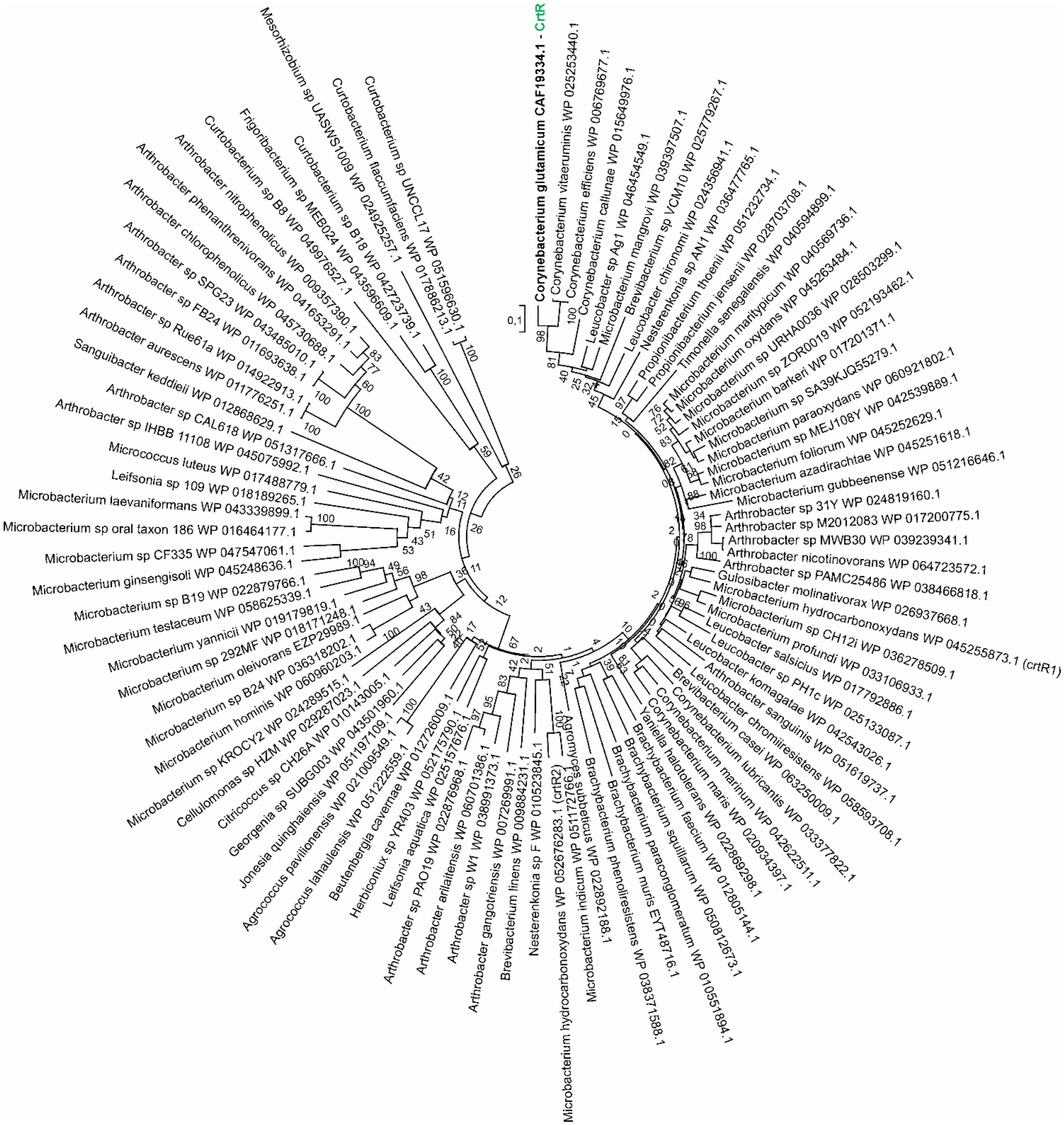

Figure S1: Phylogenetic relation of *C. glutamicum* CrtR (green) and orthologs of various bacteria. The sequence alignment and phylogenetic tree analysis were conducted with MEGA version 6 (Tamura, et al 2013) using ClustalW and the neighbor-joining method.

```
CLUSTAL O(1.2.4) multiple sequence alignment


Corynebacterium_glutamicum          MLNMQEPDKIHPAEPTLRNIYDVKTSDPKSELVDRSGMSEEDIAQIGRLMKSLASLRDVERSIGEASARYMEL
Corynebacterium_vitaeruminis        ---MQESEAKHGDESPLNNLYDVKSSDPRSELIDRSDVSPADLAQIGRLMKALANLRDTERAVAAAASRYMAL
Corynebacterium_efficiens           ---MQESETKHGDESPLNNLYDVKSSDPRSELIDRSDVSPADVAQIGRLMKALANLRDTERAVAAAASRYMAL
Corynebacterium_callunae            ---MSDPQEPQQWQAISQNLFDVDSSDPNSELVDRSSLSTEEVAQIGRVMKSLADLRNAERAVAEASEKYMKL
Leucobacter_sp._Ag1                 ---MPEAPGGGVPDPIAANLYEVDSSDPRSELVDRSGLSPEAVAQIGRLMGSLSALRDVEQRVSDASQKYMRL
Microbacterium_mangrovi             --MAEKGARAARQDSISENLYEVDSADPRSQLVDRSGLKPEEVAQIGRLMKAIAALRETEKAVAEASQRYMRL
Brevibacterium_sp._VCM10            ---MESTEAAGVHDPITENMYTGSQSSFPGDLVDSSDLSAADREQIAAVMNALARLREAERALAEASRRFMKL
Leucobacter_chironomi               ----MDHPSDETVDRISENLYRVGLKAGSSDLVDVSNVPPAAMDEIADLMNALARLREAETKLSEASRKYMKL
                                         .       :    *::        .:*:* *.:      :*. :* ::: **:.*  :. *: ::* *

Corynebacterium_glutamicum          SAPDMRALHYLIVAGNAGEVVTPGMLGAHLKLSPASVTKTLNRLEKGGHIVRNVHPVDRRAFALMVTDATRGE
Corynebacterium_vitaeruminis        SAQDMRALHYLMVAGNTGQVVTPGMLGVHLNLSPASVTKMLNRLEKGEHIIRKVHPVDRRAFALEVTQATRAE
Corynebacterium_efficiens           SAQDMRALHYLMVAGNTGQVVTPGMLGAHLNLSPASVTKMLNRLEKGEHIIRKVHPVDRRAFALEVTQATRAE
Corynebacterium_callunae            NTQDMRALHYLIVAKHQNEVVTPGMLGHYLNLSPASITKLLNRLEKGGHIVREVHPFDRRAFAIEVTPSTRTS
Leucobacter_sp._Ag1                 SAQDMRALHYLIVAKNRAELATPGMLAAHLGISAASTTKLLNRLERGGHVVRNVHPADRRAFAIEVTRETEVS
Microbacterium_mangrovi             SAVDMRALHYLIIAKHQGDVVSPGMLAGHLGISAASTTKLLNRLENGGHIVRAVHPLDRRAFAIEVTPETEAS
Brevibacterium_sp._VCM10            SEQDMRALHYLIAAKRQNAAVTPKMLSAHMSMSAASVTKLINRLERDGHVIRKLHPSDRRAYALEVTAETTRS
Leucobacter_chironomi               SEQDMRALHYLIVAKRQGDIVTPGMLAAHLRISPASTTKLLNRLERDAHIVRSMHPSDRRAFMIDITPETESS
                                    .  ********: * .    .:* **. :: :* ** ** :****.. *::* :** ****: : :*  *  .
                                    _______________________pfam12802_____________________________

Corynebacterium_glutamicum          AMRTLGKHQARRFDAAKRLTPQEREVVIRFLQDMAQELSLNNAPWLNTE----------------
Corynebacterium_vitaeruminis        AMETLGRHQARRFESAKRLTSDEREVVIRFLEDMANELSLSNAAWAEGDAGSRAAAPSAGQDVNSG
Corynebacterium_efficiens           AMETLGRHQARRFESAKRLTSEEREVVIRFLEDMANELSLSNAAWAEGDAGSRAAAPPAGQDAKSG
Corynebacterium_callunae            AMQTVGKQHAKRFQSAARLTPEEREVVIRFFEDMTQELLLEGVDWFESSSH---------------
Leucobacter_sp._Ag1                 AMQTVGRQQAKRFHAAARLTGEERETVIRFLDDMAAELSLDGADWAGGEAEHRSE-----------
Microbacterium_mangrovi             AMETVGRQQSRRFFAAARLTHDEREVVIRFLEDMAREMSLTGVDWAGDAHG---------------
Brevibacterium_sp._VCM10            ARETVGRTQARRIHAAADLTSRERDAVIRFLNGMTEELSLDNVDWTKQP-----------------
Leucobacter_chironomi               AKQTVGRQQARRFYAAARLTSEERAVVTRFLTDMASEISLTRADWANPAEAPSDS-PAADPAAE--
                                    * .*:*: :::*: :*  **  ** .* **: .*: *: *  . *
```

Figure S2: Alignment of the closest relatives of CrtR from *C. glutamicum*. The predicted HTH motif and the conserved amino acids are depicted in green. Accession numbers: *C. glutamicum* (CAF19334); *C. vitaeruminis* (WP_025253440; *C. efficiens* (WP_006769677); *C. callunae* (WP_015649976); *Leucobacter* sp. Ag1 (WP_046454549); *M. mangrovi* (WP_039397507); *Brevibacterium* sp. VCM10 (WP_025779267); *L. chironomi* (WP_024356941).

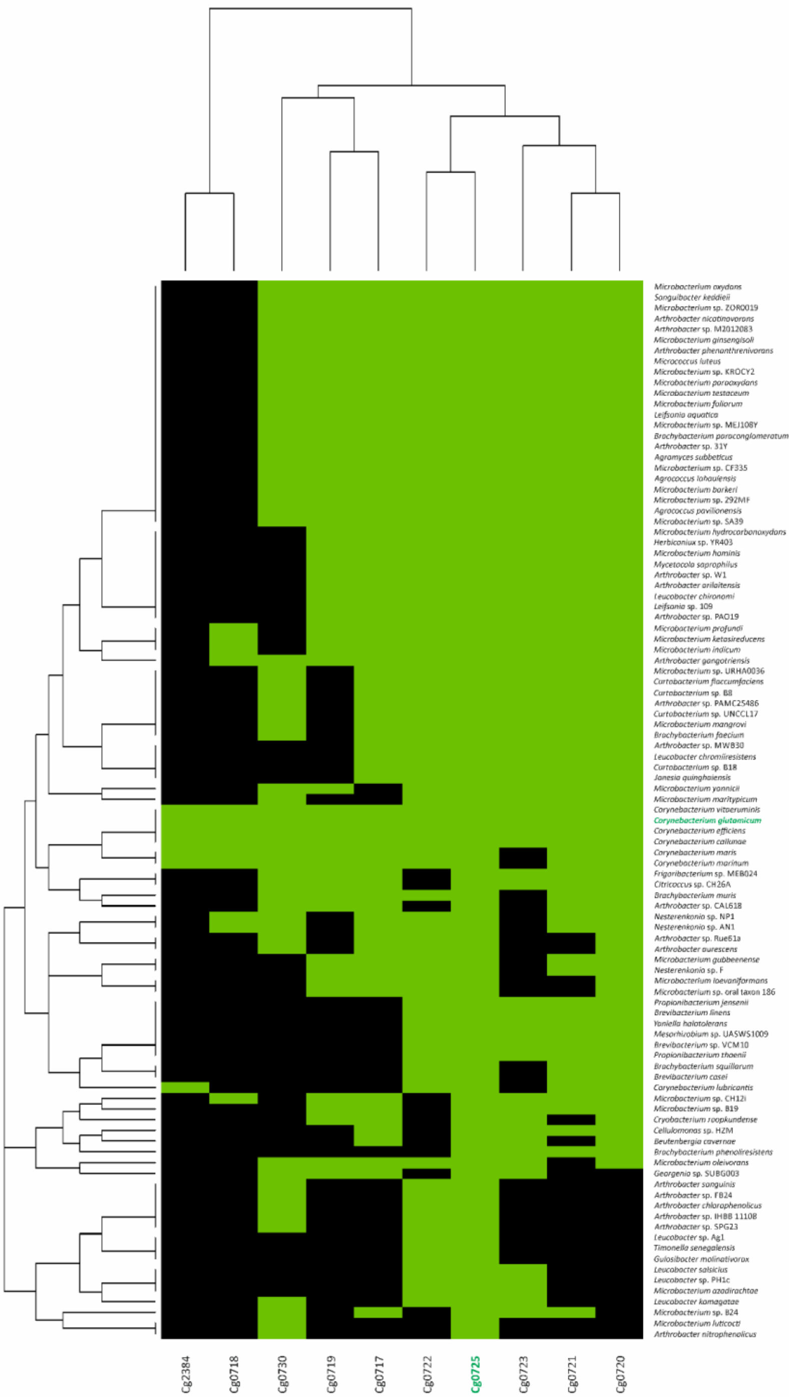

Figure S3: Hierarchical cluster analysis of CrtR and Crt proteins. A hierarchical cluster analysis was performed using BLAST results for completely sequenced organisms, possessing a CrtR homolog and at least one additional gene of the group *crtEb* (cg0717), $crtY_f$ (cg0718), $crtY_e$ (cg0719), *crtI* (cg0720), *crtB* (cg0721), cg0722, *crtE* (cg0723), *crtX* (cg0730), and *idsA* (cg2384). Only matching sequences with at least 25 % amino acid identity and an e value less than e^{-10} were subjected to the hierarchical cluster analysis (scoring the absence or presence of a *crt* homologous gene). Clustering and heatmap construction were performed via R. Colorcode: black indicates no hit with given parameters; green indicates a hit with given constraints.

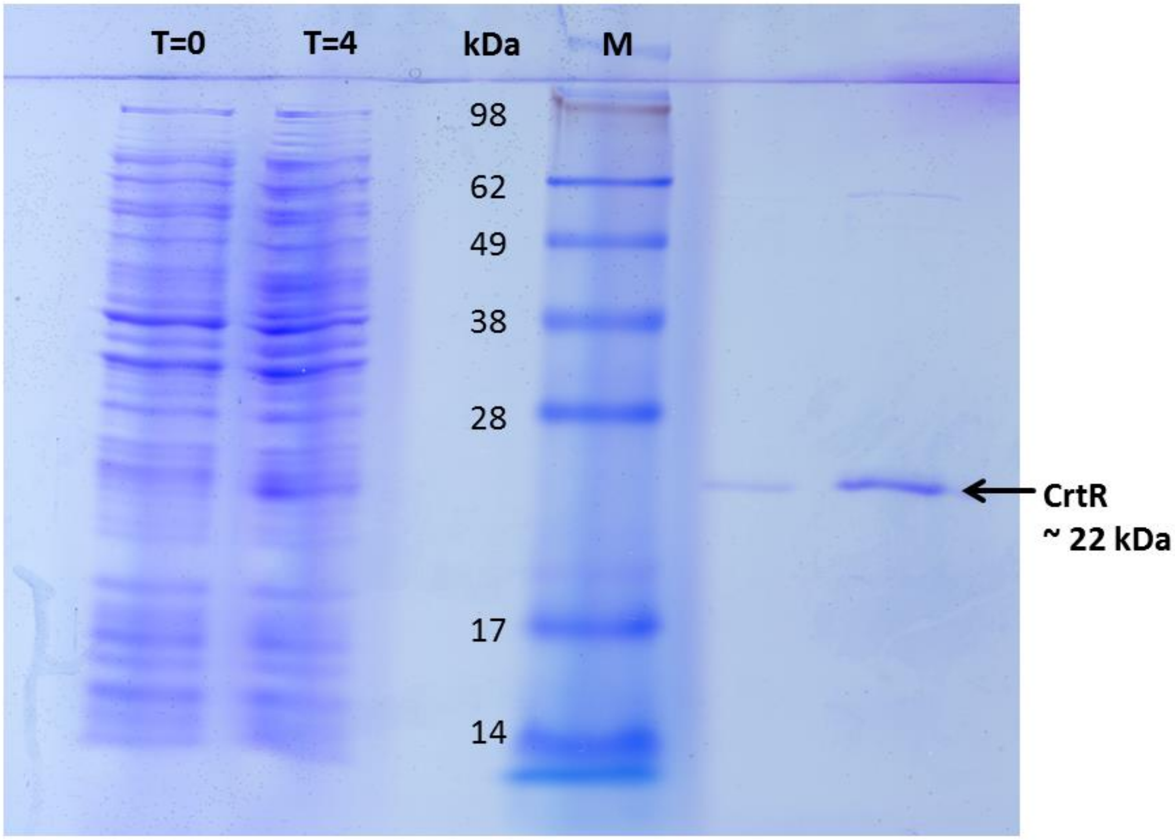

Figure S4 Sodium dodecyl sulfate-polyacrylamide gel electrophoreses of expression and purification of CrtR^{His} of *C. glutamicum* from *E. coli* BL21(DE3; pET16b-*crtR*). The gel was loaded with cell extract from *E. coli* (pET16b-*crtR*) before (lane 1) and 4 h after induction with 0.5 mM IPTG (lane 2), and two different fractions of the eluate of Ni-NTA chromatography after rebuffering on a PD-10 column (lane 4, 5). Lane 3 (M) shows protein standards SeaBlue Plus2 prestained standard (Invitrogen) containing protein of the indicated masses.

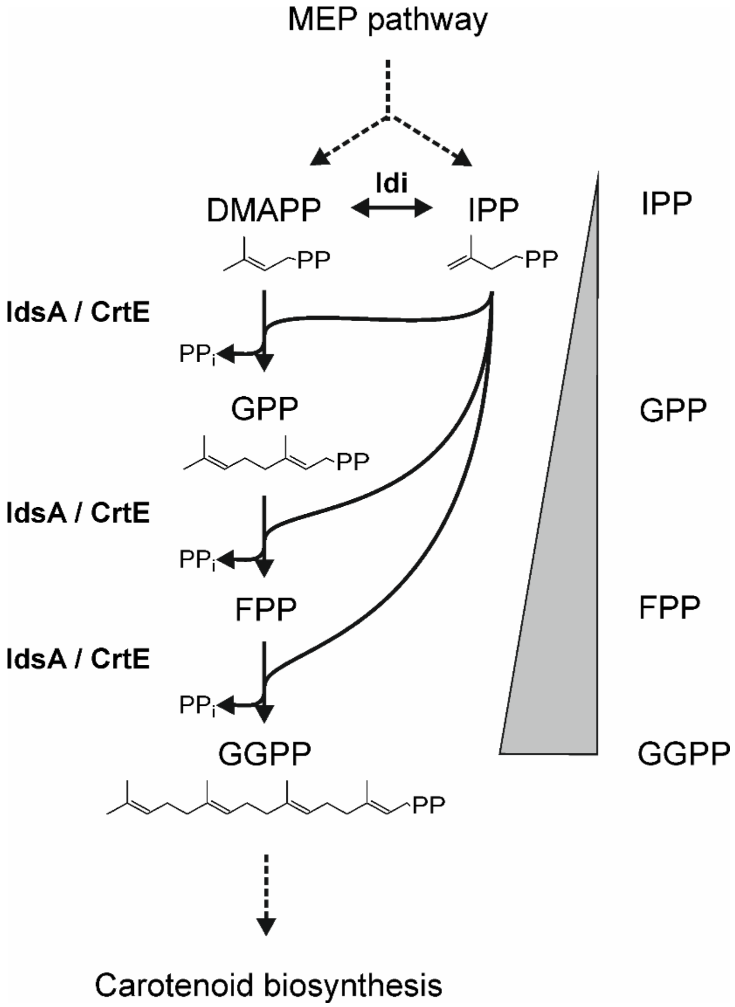

Figure S5: Isoprenoid pyrophosphate pathway in *C. glutamicum*. Dimethylallyl pyrophosphate (DMAPP), isopentenyl pyrophosphate (IPP), methylerythritol phosphate pathway (MEP), isopentenyl pyrophosphate isomerase (Idi), GGPP synthase (IdsA/CrtE), geranyl pyrophosphate (GPP), farnesyl pyrophosphate (FPP), geranylgeranyl pyrophosphate (GGPP).

a)

	growth rate [μ]
MB001(pVWEx1)	0.44 ± 0.04
MB001Δ*crtR*(pVWEx1)	0.47 ± 0.05
MB001Δ*crtR*(pVWEx1-*crtR*)	0.44 ± 0.01

b)

MB001

MB001(pVWEx1-*crtR*)

MB001Δ*crtR*

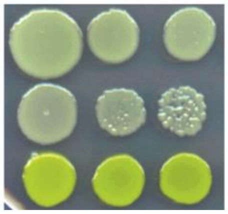

Figure S6: Growth rates and phenotypes of different *C. glutamicum* strains. Growth rates of *crtR* deletion strain MB001Δ*crtR*(pVWEx1) and the respective *crtR* overexpressing strain MB001Δ*crtR*(pVWEx1-*crtR*) in comparison to the control strain MB001(pVWEx1) in liquid CgXII minimal medium with glucose as carbon source (a) and colony color after growth of MB001Δ*crtR*, MB001(pVWEx1-*crtR*) in comparison to the parental strain MB001 on a CgXII minimal medium agar plate (b).
